# Updated Trends in Imaging Practices for Pancreatic Neuroendocrine Tumors (PNETs): A Systematic Review and Meta-Analysis to Pave the Way for Standardization in the New Era of Big Data and Artificial Intelligence

**DOI:** 10.3389/fonc.2021.628408

**Published:** 2021-07-14

**Authors:** Ephraïm Partouche, Randy Yeh, Thomas Eche, Laura Rozenblum, Nicolas Carrere, Rosine Guimbaud, Lawrence O. Dierickx, Hervé Rousseau, Laurent Dercle, Fatima-Zohra Mokrane

**Affiliations:** ^1^ Radiology Department, Rangueil University Hospital, Toulouse, France; ^2^ Memorial Sloan Kettering Cancer Center, Molecular Imaging and Therapy Service., New York, NY, United States; ^3^ Sorbonne Université, Service de Médecine Nucléaire, AP-HP, Hôpital La Pitié-Salpêtrière, Paris, France; ^4^ Surgery Department, Toulouse University Hospital, Toulouse, France; ^5^ Oncology Department, Toulouse University Hospital, Toulouse, France; ^6^ Nuclear Medicine Department, IUCT-Oncopole, Toulouse, France; ^7^ Department of Radiology, New York Presbyterian Hospital, Columbia University Vagellos College of Physicians and Surgeons, New York, NY, United States

**Keywords:** pancreatic neuroendocrine tumors (pNETs), imaging practices, meta-analysis, systematic review, computed tomogaphy, MRI, PET - positron emission tomography

## Abstract

**Purpose:**

Medical imaging plays a central and decisive role in guiding the management of patients with pancreatic neuroendocrine tumors (PNETs). Our aim was to synthesize all recent literature of PNETs, enabling a comparison of all imaging practices.

**Methods:**

based on a systematic review and meta-analysis approach, we collected; using MEDLINE, EMBASE, and Cochrane Library databases; all recent imaging-based studies, published from December 2014 to December 2019. Study quality assessment was performed by QUADAS-2 and MINORS tools.

**Results:**

161 studies consisting of 19852 patients were included. There were 63 ‘imaging’ studies evaluating the accuracy of medical imaging, and 98 ‘clinical’ studies using medical imaging as a tool for response assessment. A wide heterogeneity of practices was demonstrated: imaging modalities were: CT (57.1%, n=92), MR (42.9%, n=69), PET/CT (13.3%, n=31), and SPECT/CT (9.3%, n=15). International imaging guidelines were mentioned in 2.5% (n=4/161) of studies. In clinical studies, imaging protocol was not mentioned in 30.6% (n=30/98) of cases and only mentioned imaging modality without further information in 63.3% (n=62/98), as compared to imaging studies (1.6% (n=1/63) of (p<0.001)). QUADAS-2 and MINORS tools deciphered existing biases in the current literature.

**Conclusion:**

We provide an overview of the updated current trends in use of medical imaging for diagnosis and response assessment in PNETs. The most commonly used imaging modalities are anatomical (CT and MRI), followed by PET/CT and SPECT/CT. Therefore, standardization and homogenization of PNETs imaging practices is needed to aggregate data and leverage a big data approach for Artificial Intelligence purposes.

## Introduction

Pancreatic neuroendocrine tumors (PNETs) are relatively uncommon tumors, with an increasing incidence due to widespread use of cross-sectional imaging (1, 2). PNETs represent a heterogeneous entity, characterized by a wide variation in clinical presentation and prognosis due to tumor functional status, possible genetic context, and variable aggressiveness, making the management of PNETs highly challenging (3–6).

Medical imaging plays a critical role in guiding PNETs patients management (7, 8). Computed tomography (CT) is often the initial modality used to evaluate pancreatic lesions, mostly because of its high spatial and temporal resolution (9), and correlation with histological prognostic factors (10). Magnetic resonance imaging (MRI) also plays a major role in pancreatic tumor characterization (11, 12) and demonstrates imaging features that can be correlated with tumor aggressiveness and grade (13, 14). A wide range of molecular imaging techniques are also used in PNET patients, as Somatostatin receptor (SSTR) imaging with single photon emission tomography/CT (SPECT/CT) and positron emission tomography/CT (PET/CT) (15), ^18^F-DOPA (16) and ^18^F-FDG with PET/CT (17). Molecular imaging techniques have shown a high association with tumor grade and are critical for theranostic approaches (18–21). Increasingly, a multimodal imaging strategy, combining anatomical and molecular techniques, are leveraged for imaging-guided approaches to personalize and optimize patient management (22, 23).

PNETs present four characteristics that make imaging evaluation challenging. First, PNETs are hypervascular slow-growing tumors and therefore, limiting thus the value of using Response-Evaluation-Criteria-in-Solid-Tumors (RECIST) because tumor burden remains stable rather than decreased in patients with the best survival (24, 25). Second, tumor size measurements may vary with contrast medium injection protocols on either CT or MRI (26). Third, new targeted cytostatic agents are used in PNETs treatment and alternative imaging criteria are needed, as tumor density change on perfusion CT (25). Fourth, immune-checkpoint modulators (ICMs) are currently being evaluated in several PNETs clinical trials (27, 28). Because of their mechanisms of action, radiologists should be aware of new patterns of response and progression to immune therapies, as well as immune Related Adverse Events (iRAE) (29–34). In addition, treatment beyond progression is allowed and immune RECIST (iRECIST) criteria should be used (35). This new era of immunotherapy makes tumor response assessment in PNETs even more difficult.

One of the key concepts unique to medical imaging is that the relevance and clinical utility of information derived from imaging depends on technical imaging parameters and acquisition. Therefore, using poor quality imaging techniques in clinical routine or in scientific studies may lead to inaccurate and biased results. Imaging examinations need to be technically adequate, uniform and homogeneous, which is even more salient in imaging PNETs since a majority of PNETs are hypervascular and up to 20% of PNETs measure 2 cm or less. Therefore, CT or MRI scans without an arterial phase acquisition or thin slices drastically reduces the sensitivity of the examination (36). Molecular imaging is also sensitive to technical parameters, with optimal patient preparation, administered radiotracer activity, and acquisition time as essential elements for high-quality molecular examinations (37, 38). Thus, imaging standardization is critically important in both clinical practice and in medical research, which encompasses clinical therapeutic trials and imaging research studies (i.e. diagnostic accuracy studies, comparison of imaging techniques, etc.). With respect to clinical trials, survival assessment, therapeutic response or prognostic value of a therapeutic effect are mostly dependent on the tumor imaging response mostly based on tumor size variations assessed by medical imaging. In an effort to harmonize and standardize clinical practice, the European Neuroendocrine Tumor Society (ENETS) published consensus guidelines for the standards of care in 2009 (38), which was updated in 2017 (37) and emphasized the importance of PNETs diagnostic procedures and technical quality of imaging methods.

In order to unravel the potential “imaging databases” that exist at the international level, we have conducted an updated review on the current imaging trends in clinical practice and research, based on a systematic review and meta-analysis approach, evaluating standardization of medical imaging in international PNETs studies during the last five years. The aim of this study was to evaluate the methodology and level of standardization of imaging in the recent literature of PNETs. We have focused this review on recent literature in order to reflect updated and current practices in imaging of PNETs, especially given the growth of literature in newer imaging techniques and theranostics.

## Materials and Methods

A preliminary step was conducted before stating this study, in which we have reviewed all available literature using different international guidelines in this area. This search is summarized in **Supplementary Table 1**.

### Literature Search Strategy and Study Selection

The study protocol was developed and previously registered in PROSPERO (study number: *CRD42020168542*).

In order to review the entire recent published literature on PNETs during the last five years, a systematic search of major reference databases MEDLINE (PubMed), CENTRAL (Cochrane Central Register of Controlled Trials) and EMBASE was undertaken in December 2019, according to the Preferred Reporting Items for Systematic Reviews and Meta-analyses (PRISMA) guidelines (39). PRISMA checklist is shown in **Supplementary Table 2**.

Key search terms included “pancreatic neuroendocrine tumor/tumor/neoplasm/carcinoma”, and “islet cell adenoma/tumor”. Study selection focused on recent literature, from December 1, 2014 to December 1, 2019. Details of search terms used for each database is reported on **Supplementary Table 3**.

After removal of duplicate studies and publications including only an abstract, non-English and non-human studies were automatically excluded from the study selection, as were case reports, systematic or non-systematic reviews and meta-analysis. The bibliographic management commercial software used was EndNote X9.3.1.

### Inclusion and Exclusion Criteria

Titles and abstracts of studies were initially screened to select eligible publications, by removing studies dealing with the following topics: (1) Publications with other NETs than PNETs or non-neuroendocrine neoplasms/*Not exclusively about PNETs*; (2) Inherited syndromes and mutated population (MEN-1, VHL, NF-1); (3) Studies investigating the diagnostic value of Ultrasound (US), endoscopic-US (EUS), EUS-guided fine needle aspiration (EUS-FNA); (4) Basic science.

Then, full-text studies of eligible publications were reviewed to select all of imaging-based publications, separated into 2 subgroups: 1) imaging diagnostic accuracy studies and related studies: comparison of two imaging techniques, evaluation of prognosis value of a subtype of imaging technique; 2) clinical studies, including observational and cohort studies, experimental studies and clinical trials, for therapeutic or prognostic purposes, whose results are themselves based on therapeutic responses and survival endpoints (Progression-Free Survival PFS, Disease-Free Survival DFS, Disease Control Rate DCR, Recurrence-Free Survival RFS, Objective Response Rate ORR), fully dependent on imaging.

All studies identified by the search were screened for eligibility by two independent authors (E.P and F.Z.M), blinded to each other’s decisions. In case of disagreement, a consensus was reached after review with a third reviewer (L.D).

### Data Extraction

The two reviewers (E.P and F.Z.M) who performed the initial literature search independently extracted relevant data for each selected imaging-based publication, using a standardized form. This includes *(a)* General publication data (title, authors, journal and year of publication, country of origin); *(b)* Study design characteristics; *(c)* Demographic, clinical and pathological variables; *(d)* Type of imaging-based survival endpoint assessed (PFS, RFS, DFS, DCR, ORR) *(e)* Any imaging available data (imaging modality used, response evaluation criteria used); *(f)* Technical characteristics and acquisition parameters of each imaging modality; *(g)* Reference or mentioning of an imaging technical guideline from international NET societies. **Table 1** summarizes all extracted data. Two investigators (E.P, F.Z.M) working in duplicate independently assessed all studies. Discordances were discussed with a third reviewer (L.D) and resolved by consensus.

**Table 1:** Extracted relevant data.

**General publication data**	Title	
	Authors	
	Journal and year of publication	
	Geographical origin	
**Study design and characteristics**	Diagnostic accuracy study	
	Cohort study	
	Clinical trial (with phase)	
	Case-control study	
	Data collection method	Prospective
		Retrospective
	Comparative	
	Randomized	
	Institutional design	Monocentric
		National multicentric
		International multicentric
**Duration of study**	Time interval	Data collection start date
		Data collection end date
		Duration of time interval
**Demographic and clinical variables**	Number of patients	
	Mean age	
	Gender	Male
		Female
	Inherited syndrome	
	Metastatic disease	
**Tumor functional status**	Functional	Insulinoma
		Gastrinoma
		Glucagonoma
		VIPoma
		Other
	Nonfunctional	
**Pathologic features**	ENETS/WHO grading	G1
		G2
		G3
	TNM/UICC staging	Stage 1
		Stage 2
		Stage 3
		Stage 4
**Imaging modality**	CT	
	MRI	
	SPECT/CT	
	PET/CT	
	Available technical acquisition parameters	
	Number of equipment	
**Anatomical imaging practices**	Detailed acquisition protocol	
	Contrast agent administration	
	Use of bolus tracking	
	Slice thickness	
**Nuclear medicine imaging practices**	Time before acquisition	
	Time acquisition	
	Molecular radiotracer	^68^Ga-DOTA
		^18^F-FDG
		^18^F-DOPA
		GLP-1R
	Radiotracer dose	
**Place of imaging in clinical studies**	Type of imaging-based survival endpoint	
	Imaging response evaluation criteria	
	Mention imaging technical guidelines	

### Methodological Quality: Risk of Bias and Quality of Evidence

We assessed the risk of bias for all included studies. First, the Quality Assessment of Diagnostic Accuracy Studies 2 (QUADAS-2) tool was used without modification as provided by the QUADAS-2 group (40). This tool aims to evaluate the methodological quality applied to four “risk of bias” domains and three “concerns regarding applicability” domains (a total of 7 items to assess). Then, we also used the Methodological Index for Non-Randomized Studies (MINORS) grading score for clinical studies (41). MINORS score is a validated tool which uses eight graded questions for non-comparative studies. We judged each domain as presenting low, high, or unclear risk of bias by using a numeric score: each item can be scored as 0 (not reported), 1 (reported but inadequate) and 2 (reported and adequate). Ideal global score varies from 16 for non-comparative studies and 24 for comparative ones.

### Statistical Analysis

Analyses were conducted using Microsoft Excel (v2019, Microsoft, USA, 2019) and open-source R software (version 3.6.2; R Foundation for Statistical Computing, Vienna, Austria). A p-value less than 0.05 was considered to indicate statistical significance (α=0.05).

## Results

### Identification and Selection of Studies

The literature search yielded 9982 studies. Following the removal of duplicates, 6509 studies remained. Among them, 4189 records including only an abstract (n=149), non-English (n=293) and non-human studies (n=2115) were automatically excluded from the study selection, as were case reports (n=1058), systematic or non-systematic reviews and meta-analysis (n=574). Afterwards, 2320 studies were screened on the basis of title and abstract. Among them, 1846 were excluded: studies not exclusively dealing with PNETs (n=951), inherited syndromes (n=133), studies evaluating ultrasound (US) (n=250), basic science studies (n=317), and unrelated studies (n=195).

**Figure 1:**
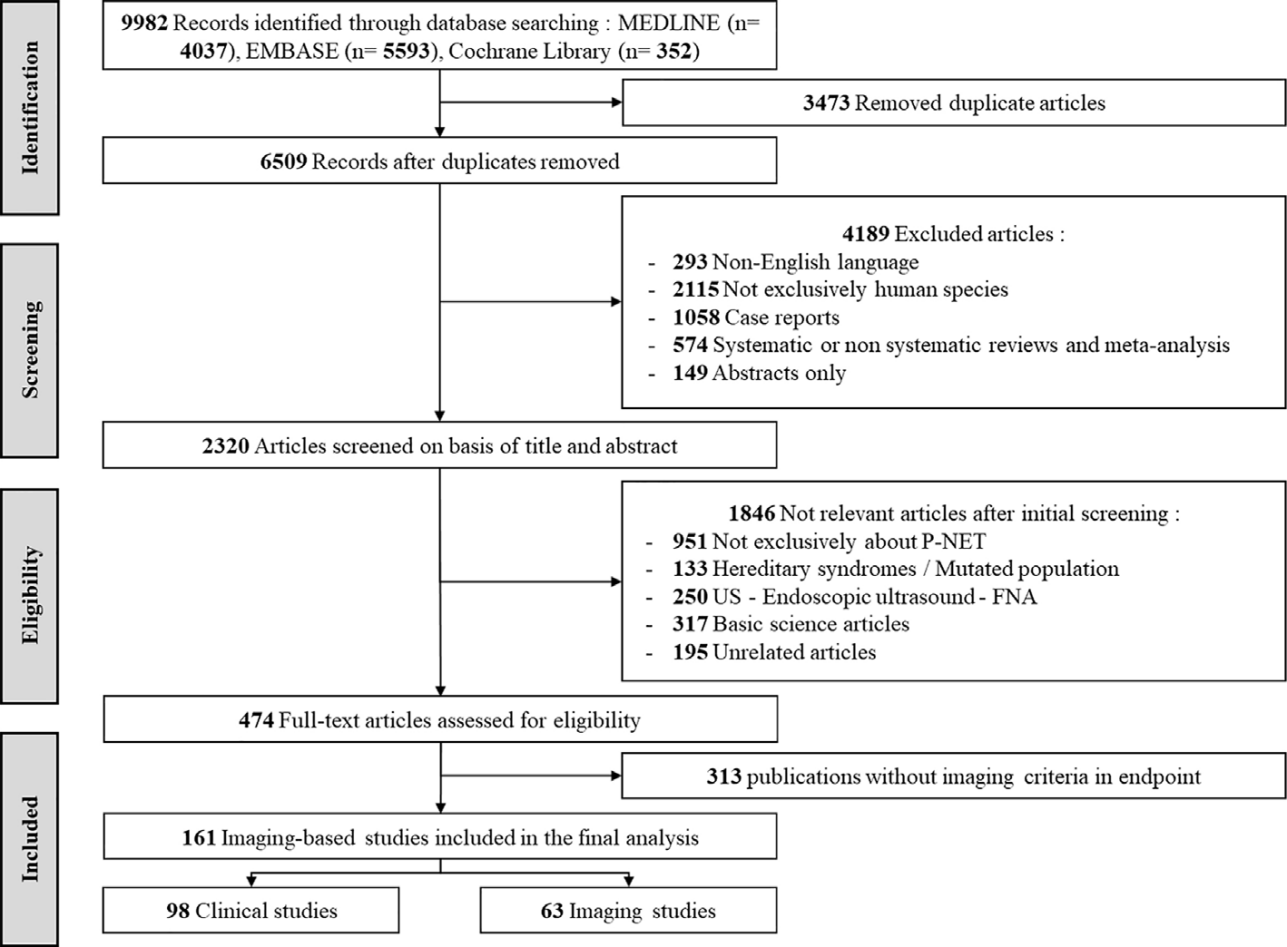
PRISMA flow chart of literature search and study selection process.

474 publications were included and fully reviewed, of which 161 were identified as imaging-based studies and included in the final analysis: 63 *imaging studies* on diagnostic accuracy studies and related studies and 98 *clinical studies* based on therapeutic responses and survival endpoints, fully dependent on imaging. The PRISMA flow chart of literature search and study selection process is shown in **Figure 1**.

### Study Characteristics

**Figure 2:**
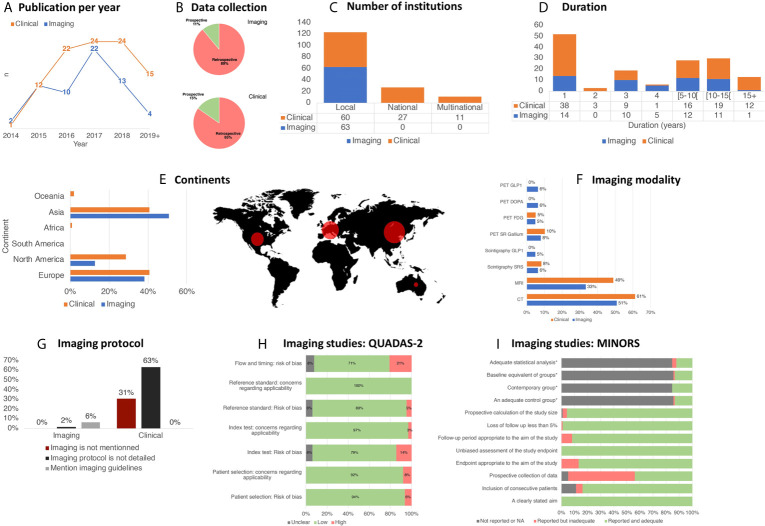
Overview of trends in imaging practices for PNETs.

Between December 2014 and December 2019, the average [range] annual number of publications was 12.6 [2;22] for imaging studies and 19.6 [1;24] for clinical studies (**Figure 2A** **)**. Sixty-three imaging studies (diagnostic accuracy studies and related studies) and ninety-eight clinical studies have been identified. Eighty-nine percent (56/63) of the imaging studies, and eighty-five percent (83/98) of clinical ones, were retrospective (**Table 2**, **Figure 2B**). The most common study design was retrospective cohorts (n=84, 85.7%). Only 3.2% (n=2) of imaging publications and 4.1% (n=4) of clinical publications were randomized. Similarly, a minority of the studies was comparative: 14.3% (n=9) of imaging publications and 15.3% (n=15) of clinical publications.

**Table 2:** Study characteristics per subgroup.

Studies characteristics		Imaging studies (n=63)	Clinical studies (n=98)	*P* value
Data collection	Prospective (%)	7/63 (11.1%)	15/98 (15.3%)	0.602
	Retrospective (%)	56/63 (88.9%)	83/98 (84.7%)	
Design	Diagnostic accuracy studies (%)	63/63 (100%)	–	N.A
	Cohort (%)	–	84/98 (85.7%)	N.A
	Clinical trial (%)	–	13/98 (13.3%)	N.A
Method	Randomization (%)	2/63 (3.2%)	4/98 (4.1%)	1
	Comparative (%)	9/63 (14.3%)	15/98 (15.3%)	1
Institutional design	Monocentric (%)	60/63 (95.2%)	60/98 (61.2%)	**<0.001**
	National multicentric (%)	3/63 (4.8%)	27/98 (27.6%)	
	International multicentric (%)	–	11/98 (11.2%)	
Blinding	Non-blinded (%)	3/63 (4.8%)	91/98 (92.9%)	**<0.001**
	Simple blinded (%)	60/63 (95.2%)	5/98 (5.1%)	
	Double blinded (%)	–	2/98 (2.0%)	

The bold values represent significant values.

A majority of imaging studies (n=60, 95.2%) were performed only in a single institution while 4.8% (n=3) were multi-institutional studies from a single country. Strikingly, no studies were international collaborations. Range of multi-institutional from a single country studies upon clinical studies was higher (27.6% (n=27)), and 11.2% (n=11) were international studies (p=<0.001) (**Figure 2C**). In addition, 92.9% (n=91) of clinical studies were non-blinded, against only 4.8% (n=3) for imaging studies (p=<0.001) (**Table 2**).

The mean duration of retrospective studies (time interval chosen in the database used) was 5.7 years [0;27] (**Figure 2D** **)**. Moreover, 71.4% (n=70/98) of clinical studies started before 2005, while 53.9% (n=34/63) of imaging studies used data collected after 2005 (p=0.007).

Finally, the geographical origin per continent of the populations studied is firstly represented by Asia (n=72, 41.1%), followed by Europe (n=64, 36.3%), and North America (n=36, 20.6%) (**Figure 2E**).

### Demographic, Clinical and Pathological Variables

In this study, 19852 patients were included, with 15728 patients in “clinical studies” and 4124 patients in “imaging studies. The mean [SD] number of patients was lower in imaging studies than clinical studies: 65.5 [52.7] and 160.5 [345.3] respectively (p=0.032). For imaging studies, 51.8% of included patients were female, with a mean (SD) age of 49.4 (15.9). In clinical studies, 49.1% were female with a mean (SD) age of 53.4 (13.0). All pathological variables, especially the tumor functional status, type of PNET if functional, rate of mutated population and metastatic disease, ENETS/WHO grading and TNM/UICC staging are summarized in **Table 3**.

**Table 3:** Demographic, clinical and pathological variables per subgroup.

	Imaging studies (n=63)	Clinical studies (n=98)	*P* value
**Number of patients**	65.5 (52.7)	160.5 (345.3)	**0.032**
**Age**	49.4 (15.9)	53.4 (13.0)	0.077
Gender*			
Male	29.8 (27.3); 48.2%	79.7 (179.0); 50.9%	**0.03**
Female	32.1 (28.8); 51.8%	76.9 (166.7); 49.1%	**0.036**
**Tumor functional status**			
Functional	9.9 (18.4)	20.4 (52.3)	0.129
*Insulinoma*	6.3 (13.0)	7.9 (27.5)	0.657
* Gastrinoma*	1.3 (5.8)	3.2 (15.8)	0.359
* Glucagonoma*	0.4 (0.8)	1.3 (6.8)	0.269
* VIPoma*	0.1 (0.2)	0.8 (3.0)	0.062
* Other functional PNET*	0.2 (1.0)	0.6 (1.9)	0.12
Nonfunctional	22.9 (43.3)	100.1 (295.7)	**0.042**
Not available	1.4 (11.3)	0.9 (4.8)	0.712
**Inherited syndrome**	2.6 (8.3)	4.2 (11.2)	0.326
**Metastatic disease**	9.4 (17.3)	43.3 (124.5)	**0.034**
**Pathologic features**			
ENETS/WHO grading			
* G1*	33.1 (36.8)	54.1 (89.1)	0.077
* G2*	18.0 (17.9)	38.8 (50.1)	**0.003**
* G3*	3.9 (5.6)	8.0 (11.7)	**0.009**
* Not available*	0.5 (1.7)	34.0 (278.9)	0.342
TNM/UICC staging			
* Stage 1*	6.1 (14.9)	26.5 (148.4)	0.28
* Stage 2*	4.1 (13.4)	15.6 (64.6)	0.166
* Stage 3*	1.7 (4.6)	6.6 (18.8)	**0.047**
* Stage 4*	2.3 (6.1)	19.1 (121.6)	0.275
* Not available*	0.1 (0.5)	0.6 (3.0)	0.128

Data are expressed as: mean (SD). *data are expressed as: mean (SD); percentage.

The bold values represent significant values. SD Standard deviation, ENETS European Neuroendocrine Tumor Society, WHO World Health Organization, TNM Tumour Node Metastasis, UICC Union for International Cancer Control.

### Recent Trends in PNET Imaging

The two imaging modalities most frequently used in the recent PNET literature are CT and MRI representing 57.1% (n=92/161) and 42.9% (n=69/161) of studies, respectively. Nuclear medicine imaging was less frequently used with 19.3% (n=31/161) of studies utilizing PET/CT and 9.3% (n=15/161) utilizing planar scintigraphy and/or SPECT/CT. No significant difference was observed between imaging and clinical studies. Detailed repartition of imaging modalities per subgroup is illustrated in **Figure 2F**.

### Standardization of Practice: Reporting of Imaging Technical Parameters

In imaging studies, available information on imaging methods, examination protocol and technical details for each imaging modality were collected and summarized in **Table 4**. With respect to CT, most studies reported a detailed imaging acquisition protocol (93.8%), with almost all studies using multiphase contrast-enhanced acquisition, except one reporting single phase acquisition. CT slice thickness parameters were available in 75.0% of studies, with a mean slice thickness of 2.6 mm. Contrast administration details were reported in most studies (81.3%), with 56.3% using contrast bolus tracking. Majority of CT studies were performed on 2 or more different types of CT-equipments (68.8%).

**Table 4:** Imaging methods, examination protocols and technical details for each imaging modality.

A. Anatomical imaging		
Modality	CT	MRI
**Number of studies per modality** (%)	32/63 (50.8%)	21/63 (33.3%)
**Detailed acquisition protocol** (%)	30/32 (93.8%)	20/21 (95.2%)
Multiphase contrast-enhanced acquisition (%)	29/30 (96.7%)	16/20 (80.0%)
Single-phase acquisition (%)	1/30 (3.3%)	3/20 (15.0%)
T1-weighted imaging	–	19/21 (90.5%)
T2-weighted imaging	–	19/21 (90.5%)
Diffusion-weighted imaging	–	16/21 (76.2%)
**CT-slice thickness available information** (%)	24/32 (75%)	17/21 (81.0%)
**CT-slice thickness** (mm) (mean ± SD)	2.6 ( ± 2.0)	3.1 ( ± 1.0)
**Details of the contrast agent administration** (%)	26/32 (81.3%)	14/21 (66.7%)
**Bolus tracking** (%)	18/32 (56.3%)	–
**Available technical acquisition parameters** (%)	21/32 (65.6%)	17/21 (81.0%)
**Number of CT/MR-system used**		
One equipment (%)	4/32 (12.5%)	10/21 (47.6%)
Two or more equipments (%)	22/32 (68.8%)	10/21 (47.6%)
Not available (%)	6/32 (18.8%)	1/21 (4.8%)
**Magnetic Field Strength (Tesla)**		
1.5 T-system (%)	–	7/21 (33.3%)
3.0 T-system (%)	–	6/21 (28.6%)
1.5 and 3.0 T-system (%)	–	7/21 (33.3%)
Not available (%)	–	1/21 (4.8%)
**B. Molecular imaging**		
**Modality**	**SPECT**	**PET**
**Number of studies per modality** (%)	7/63 (11.1%)	16/63 (25.4%)
**Molecular radiotracer**		
^68^Ga-DOTA (%)	4/7 (57.1%)	5/16 (31.3%)
GLP-1R (%)	3/7 (42.9%)	4/16 (25%)
^18^F-FDG (%)	–	3/16 (18.6%)
^18^F-DOPA (%)	–	4/16 (25%)
**Radiotracer dose**		
^68^Ga-DOTA (MBq) (mean ± SD)	227.1 ( ± 100.3)	145.2 ( ± 33.1)
GLP-1R (MBq) (mean ± SD)	299.8 ( ± 381.2)	88.8 ( ± 11.4)
18F-FDG (MBq) (mean ± SD)	–	328.1 ( ± 106.3)
18F-DOPA (MBq) (mean ± SD)	–	263.8 ( ± 18.9)
**Time before acquisition (min) (mean ± SD)**	1181 ( ± 972.8)	57.5 ( ± 42.6)
**Time acquisition**		
Not available (%)	6/7 (85.7%)	–
Available (%)	1/7 (14.3%)	–
**Number of systems used**		
One equipment (%)	5/7 (71.4%)	11/16 (68.8%)
Two or more equipments (%)	–	4/16 (25%)
Not available (%)	2/7 (28.6%)	1/16 (6.3%)

**A:** imaging methods, examination protocol and technical details for each imaging modality: anatomical imaging. CT Computed Tomography, MRI Magnetic Resonance Imaging.

**B:** imaging methods, examination protocol and technical details for each imaging modality: molecular imaging, SPECT Single-Photon Emission Computed Tomography, PET Positron Emission Tomography, SSTR Somatostatin Receptor, 18F-FDG Fluorodeoxyglucose, 18F-DOPA Fluorodeoxyphenylalanine, GLP-1R Glucagonlike Peptide-1 Receptor, SD Standard Deviation.

For MRI, most studies reported an imaging acquisition protocol (95.2%, n=20/21), with almost all studies acquiring multiphase contrast-enhanced images (80%, n=16/20), T1- and T2-weighted images (90.5%), and majority of studies obtaining DWI images (76.2%). MRI slice thickness parameters were available in 81.0% of studies, with a mean slice thickness of 3.1 mm. Details of contrast administration were reported in 66.7% of studies. Among studies in which MRI scanner details were reported (n =20/21), half of them were performed on one single MRI scanner, while other half were performed on 2 or more different scanners. For magnet field strength, 33.3% were performed on a 1.5 T system, 28.6% on a 3.0 T system, and 33.3% used both 1.5 and 3.0 T systems.

For PET/CT imaging, the most common radiotracer studied in PNETs is ^68^Ga-DOTA (31.3%), followed by the GLP-1R agonist (25%), ^18^F-DOPA (25%), and ^18^F-FDG (18.6%). Most studies were performed on one scanner (68.8%, n=11/16), while the rest were performed on 2 or more scanners (25%, n=4/16), with 1 study not reporting scanner details. For SPECT/CT, 57.1% of studies evaluated SSTR radiotracers, while 42.9% studied GLP-1R. Most studies reported scanner details (71.4%, n=5/7), with all of them performing it on a single scanner.

There was significant heterogeneity regarding the reporting of imaging modalities and imaging acquisition protocols (**Figure 2G**). For example, 30.6% (n=30/98) of clinical studies did not describe which imaging modalities were used, in contrast to imaging studies, which specified the imaging modality in 100% of cases (n=63/63) (p<0.001). Additionally, 63.3% of clinical studies (n=62/98) reported the imaging modalities used, however, no details on the imaging protocol were reported, while only one imaging study did not report the specific imaging protocol used (1.6%, p<0.001). In only 11.2% (n=11) of clinical studies, injection phase was specified, and in 4.1% (n=4), the multiphase injection phase was clearly specified.

Studies rarely mentioned international guidelines with no clinical study and only four (6.3%) imaging studies referring to guidelines, all of them exclusively referencing ENETS 2009 guidelines (n=4) (16, 42–44) with no studies referencing the most updated 2017 ENETS technical guidelines (37).

### Methodological Quality: Risk of Bias Assessment

#### Imaging Studies

For imaging studies (QUADAS-2: **Figure 2H**), no study was considered to be at low risk of bias for all domains. In 6.3% of included studies, a high risk of bias for patient selection was due to non-consecutive or random enrollment. Regarding the patient flow and timing, 20.6% of imaging studies used a combination of histopathologic findings and clinical follow-up in reference standard, introducing a high risk of bias. Lastly, in 4.8% of studies, a high risk of bias was recorded due to a non-blinded nature of reference standard assessment of imaging test results.

#### Clinical Studies

Using MINORS index for clinical studies (**Figure 2I**) allowed highlighting the fact that the main bias was introduced by the lack of prospective collection of data in 56.1% of the time. The second major bias was the lack of information on the consecutive nature of the inclusion of patients (16.3%). Of note, only a small proportion of the clinical studies was comparative (13.3%, n=15/98), which precluded the possibility to evaluate the four additional criteria (adequate control group, contemporary groups, baseline equivalence of groups, adequate statistical analyses).

## Discussion

Medical imaging plays a decisive role in PNETs management, a highly challenging disease (22), and is represented by a large panel of imaging tools available to physicians. With a purpose of standardizing practices, ENETS 2017 guidelines emphases on the need for technical quality of imaging methods (37, 38, 45). To optimize treatment strategies, it is often necessary to combine data from several centers in clinical therapeutic trials. In the new era of big data and artificial intelligence, harmonization of imaging practices is especially important to find relevant imaging biomarkers. This also explains the importance of assessing practice heterogeneity, in order to unravel the potential “imaging databases” that exist in this field. Based on this unmet need, the first objective of this systematic review was to assess the level of standardization of imaging practices in the recent PNETs literature.

In this study, we demonstrated the existence of a significant lack of standardization and homogenization of methodological imaging practices in the recent PNETs literature. Study selection resulted in 161 imaging-based manuscripts and allowed the creation of two different sub-groups of publications in the final analysis: 63 imaging studies and 98 clinical studies.

The choice of studying each sub-group separately can be explained by our assumptions about the differences in conduction of each type of studies. In imaging studies subgroup, we expected to have all the necessary details because the purpose of these studies is to evaluate diagnostic accuracy. We wanted to assess the degree of homogeneity and compare this information with the international guidelines. For clinical studies in which the therapeutic evaluation is obtained by radiological assessment, we have hypothesized a very small amount of technical details since clinical outcomes were the primary endpoints.

Our study is the first to evaluate imaging standardization in PNETs. Beyond the overall lack of methodological standardization and homogenization, six key concepts were identified in this study.

First, overall methodology quality remains suboptimal. Indeed, the vast majority of the studies was retrospective (n=139/161; 86.3%) and non-randomized (n=155/161; 96.3%). However, there was a significant difference between the two subgroups in terms of institutional design, with multicentric nature in 38.8% of clinical studies, versus less than 5% of imaging studies (p=<0.001). This point may indicate that clinical studies are generally more qualitative, in a methodological point of view. At the opposite, clinical studies were mostly non-blinded, against only 4.8% of imaging studies (p=<0.001), making thus imaging diagnostic accuracy studies’ evaluation methodologically valid. While clinical studies are prospective and multicentric, there is limited reporting of and lack of standardization of the imaging acquisition in these studies, which may lead to heterogeneous image quality. Imaging studies have more homogeneous and better described imaging techniques, but the level of evidence is limited by the fact that studies are monocentric and retrospective.

Second, there is a mismatch between types of data used for clinical or imaging studies. While 71.4% of clinical studies started collecting data before 2005 (n=70/98), 53.9% (n=34/63) of imaging studies used data collected after 2005. This point highlights the possible difference between results obtained with clinical studies as compared to imaging ones. Indeed, molecular imaging in the field of NET has been extensively developed this last decade, vastly improving the performance of imaging techniques through more accurate methods, such as Ga-68 DOTATATE PET/CT imaging (46, 47). The two most common imaging modalities reported in the recent PNETs literature are CT and MRI, despite significant progress in nuclear medicine imaging, with the advent of newer high-performance PET radiotracers and its increased availability. Therefore, we predict there will be a future rebalancing in the partition of different PNETs imaging modalities.

Third, geographical distribution of populations in the current literature shows a lack of representation of patients from South America, Africa and Oceania, although the prevalence of PNETs in these parts of the world is not different (48). In other terms, international societies need to encourage research in these countries in order to obtain worldwide results, and better homogenize PNET patients’ management, both in clinical routine and for research purposes.

Fourth, imaging procedures are described more frequently and in better detail in imaging studies than in clinical studies, even in large multicentric international clinical studies. Moreover, the radiological assessment is also of better quality in imaging studies, with a significantly higher rate of blinded assessors. In 90.8% of clinical studies, imaging assessment was not clearly stated. Paradoxically, in this study, multicentric international studies, which are supposed to be methodologically better, presented lower quality in terms of radiological methodology. This can be explained by a lack of standardization between each center. For instance, RADIANT-3, a large prospective, randomized, phase 3 clinical trial, published in 2011, demonstrated improvement of everolimus in progression-free survival (PFS) compared with placebo for patients with advanced PNETs (49). Contrasting with the vast majority of the recent clinical studies analyzed, imaging technical details were fully described in their supplementary materials. Since progression-free survival is in part an imaging-based clinical endpoint, this fact confirms that this study is methodologically correct in terms of technical quality and imaging protocol and has a high evidence-based value.

Fifth, adherence to international guidelines is very low in the included studies, as shown by low rates of reference to international imaging technical guidelines (2.5% (n=4/161) of all selected studies). In these 4 cases (published between 2015 and 2018), ENETS 2009 technical guidelines were mentioned. We noticed that ENETS 2017 technical guidelines were never mentioned in the 102 selected articles published since 2017, although it was the most recent and detailed guidelines.

Last, there was a lack of imaging quality assessment tools. Indeed, many tools and indexes are available for methodological quality evaluation of studies and assessment of risks of bias. We have chosen to use MINOR and QUADAS-2 because of their simplicity and their reliability, as demonstrated by the rigorous and evidence-based process to develop these tools. However, neither of these tools were specifically designed to assess how standardized imaging procedures are performed, which can be essential in some areas. Therefore, it seems important that future work focuses on a methodological quality assessment tool that incorporates the evaluation of how imaging techniques are performed.

Based on a systematic review and meta-analysis approach, using strict inclusion criteria, we applied state-of-the-art methodology in this research. We have chosen to restrict our search strategy to the last five years. Indeed, we focused on imaging technical parameters, a field of medicine and technology that is constantly evolving and changing. For example, thin CT sections were not routinely systematic before 2009 ENETS technical guidelines.

These results showed the difficulty of pooling all data for a big data approach. Qualitative assessment of potential “imaging databases”, theoretically accessible to Datamining using AI in recent PNETs literature shows an excessive data heterogeneity. This is exacerbated by the use of many different machines and equipment, which increases input data variability. Initiatives like the EARL FDG PET/CT accreditation program provide a way to limit the data heterogeneity and facilitate multicenter research projects with accurate and reproducible imaging data.

Results expressed in this study might have major implications for clinicians, researchers, and guideline committees. Clinical decisions should be based on the best available imaging technique, using rigorously the recommended technical properties for each technique. A non-optimal imaging acquisition or reconstruction should be repeated before taking any clinical decision.

Similarly, precision should be requested in imaging-based studies. In addition, as a quality guarantee, affirmation of the use of imaging examinations in accordance with reference guidelines should be at least mentioned before envisioning any future publication. Another approach to improve practices would be to modify prospective databases from which a majority of studies collect their information. Technical imaging data, radiological protocols and acquisition methods should be mentioned, and only patients who have benefited from appropriate imaging examinations in accordance with international guidelines should be included. A proposal to expand these databases to include imaging technical information would also allow better selection of patients with technically correct imaging.

Herein, this systematic review of the recent literature on PNETs, with a special emphasis on imaging, demonstrates the lack of rigorous reporting and standardization of imaging techniques in clinical practice and research. Indeed, a clear gap in imaging information in clinical studies was demonstrated, particularly for types of modalities used, radiological protocol applied, and imaging assessment. This lack of information seems more intriguing, when it comes to clinical studies whose results are mainly based on radiological evaluation. Even when technical details were available in imaging studies, there is a significant heterogeneity of practices and a lack of references to established international guidelines. This non-uniformity makes it difficult to envision a pooled use of data for AI datamining and big data purposes since AI needs absolute homogeneity and standardization of clinical practices, that will perhaps allow identifying new biomarkers for treatment effectiveness, and thus a higher optimization of PNETs patients’ management.

## Data Availability Statement 

The original contributions presented in the study are included in the article/**Supplementary Material**. Further inquiries can be directed to the corresponding author.

## Author Contributions

The study was designed, directed and coordinated by F-ZM and LD. EP conducted the study, wrote the manuscript and designed the figures. LD and RY performed the statistics LR and LOD analyzed nuclear medicine data. TE, RY, NC, RG, and HR reviewed the manuscript. All authors contributed to the article and approved the submitted version.

## Conflict of Interest

The authors declare that the research was conducted in the absence of any commercial or financial relationships that could be construed as a potential conflict of interest.

## Supplementary Material

Supplementary Table 1 Available international guidelines for PNETs technical imaging.

Supplementary Table 2 PRISMA 2009 checklist

Supplementary Table 3 Search strategies.
