# Supplementary material for: Updated Trends in Imaging Practices for Pancreatic Neuroendocrine Tumors (PNETs): A Systematic Review and Meta-Analysis to Pave the Way for Standardization in the New Era of Big Data and Artificial Intelligence
Source: Front Oncol. 2021 Jul 14;11:628408. doi: 10.3389/fonc.2021.628408 (PMC8316992; doi:10.3389/fonc.2021.628408)
Supplement: Supplementary Table 1 — Available international guidelines for PNETs technical imaging. [file Table_1.docx]

| **International NET societies** | **European Neuroendocrine Tumor Society (ENETS)** | | | **North American Neuroendocrine Tumor Society (NANETS)** | | |
| --- | --- | --- | --- | --- | --- | --- |
| Title | ENETS Consensus Guidelines for the Standards of Care in Neuroendocrine Tumors: radiological examinations | ENETS Consensus Guidelines for the Standards of Care in Neuroendocrine Tumors: somatostatin receptor imaging with (111)In-pentetreotide | ENETS Consensus Guidelines for the Standards of Care in Neuroendocrine Tumors: Radiological, Nuclear Medicine & Hybrid Imaging | NANETS treatment guidelines: well-differentiated neuroendocrine tumors of the stomach and pancreas | Consensus guidelines for the management and treatment of neuroendocrine tumors. Pancreas | Appropriate Use Criteria for Somatostatin Receptor PET Imaging in Neuroendocrine Tumors |
| Authors | Sundin et Al. | Kwekkeboom et Al. | Sundin et Al. | Kulke et Al. | Kulke et Al. | Hope et Al. |
| Year of publication | 2009 | 2009 | 2017 | 2010 | 2013 | 2018 |
| Journal name | Neuroendocrinology | Neuroendocrinology | Neuroendocrinology | Pancreas | Pancreas | The Journal Of Nuclear Medicine |
| Volume, Issue, and Page Range | 90(2):167‐183 | 90(2):184‐189 | 105(3):212‐244 | 39(6):735‐752 | 42(4):557‐577 | 59(1):66‐74 |
| **Relevant imaging modalities** | | | | | | |
| **CT** | **🗸** | **🗴** | **🗸** | **🗸** | **🗸** | **🗴** |
| **MRI** | **🗸** | **🗴** | **🗸** | **🗸** | **🗸** | **🗴** |
| **SPECT/CT** | **🗴** | **🗸** | **🗸** | **🗸** | **🗸** | **🗴** |
| **PET/CT** | **🗴** | **🗴** | **🗸** | **🗸** | **🗸** | **🗸** |

**Appendix: supplementary materials**

**Supplementary Table 1**

| **Computed Tomography (CT)** | | | | | | |
| --- | --- | --- | --- | --- | --- | --- |
| Slice thickness and examination parameters | Acquisition: ≤3 mm Transverse reconstruction: 2-3 mm (30-50% overlap) pitch of 1.25–1.5 | **-** | Acquisition: ≤1 mm Transverse reconstruction: 2-3 mm (30-50% overlap) pitch of 1.25–1.5 | Thin slice CT | Thin slice CT | **-** |
| Image post-processing | Multiplanar reconstructed images (MPR) 3D maximum intensity projections (MIP)  Volume-rendering technique (VRT) |  | Multiplanar reconstructed images (MPR) 3D maximum intensity projections (MIP)  Volume-rendering technique (VRT) | **-** | **-** |  |
| Patient preparation | Drinking approximately 800 ml of tap water (without addition of an iodine-based contrast medium) No solid food for the previous 12h Optional anti-peristaltic agent |  | Drinking approximately 800 ml of tap water (without addition of an iodine-based CM) No solid food for the previous 12h Optional anti-peristaltic agent | **-** | **-** |  |
| Precautions | Impaired renal function CM-related adverse reaction Diabetes |  | Impaired renal function CM-related adverse reaction Diabetes | **-** | **-** |  |
| Imaging protocol | Bolus-tracking technique Non-enhanced examination Early arterial phase Late arterial phase Portal-venous phase |  | Bolus-tracking technique Non-enhanced examination Early arterial phase Late arterial phase Portal-venous phase | Rapid intravenous bolus Arterial phase Venous phase | **-** |  |
| Intravenous contrast medium injection | Dose : 1.5–2 ml/kg body weight (maximum 180 ml) Concentration : 300–350 mg/ml non-ionic low- or iso-osmolar contrast material  Injection rate : ≥5 ml/s using a power injector |  | Dose : 1.5–2 ml/kg body weight (maximum 180 ml) Concentration : 300–350 mg/ml non-ionic low- or iso-osmolar contrast material  Injection rate : 3-5 ml/s using a power injector | **-** | **-** |  |

| **Magnetic Resonance Imaging (MRI)** | | | | | | |
| --- | --- | --- | --- | --- | --- | --- |
| Magnetic Field Strength (Tesla) | ≥ 1,5 Tesla | **-** | ≥ 1,5 Tesla | **-** | **-** | **-** |
| Slice thickness and examination parameters | 3 mm (< 5 mm) Use of a phased-array torso coil |  | 3 mm (< 5 mm) Use of a phased-array torso coil | **-** | **-** |  |
| Patient preparation | Optional distension of the stomach and duodenum with paramagnetic agent No solid food for the previous 6h Anti-peristaltic agent |  | Optional distension of the stomach and duodenum with paramagnetic agent Anti-peristaltic agent | **-** | **-** |  |
| Precautions | Impaired renal function Diabetes |  | Impaired renal function Diabetes | **-** | **-** |  |
| Imaging  protocol | 1. Anatomical / morphological sequences: FS axial T1, FS T2 (optional: FS axial T1 IP/OP) 2. Dynamic contrast enhancement: 30s - 70s - 120s - 3-5 min 3. Additional sequences for pancreatic evaluation: CP IRM Coronal radiated T2-weighted thickslice (25 mm) sequences, T2 thin slices MRCP with 3D acquisition |  | 1. Anatomical / morphological sequences: axial T2, coronal T2 (optional Fat Sat et SPIR), axial T1, axial T1 FS, In-phase and out-of-phase sequences 2. DWI : with low, intermediate and high b-values and ADC map 3. Dynamic contrast enhancement: 30s - 70s - 120s - 3-5 min 4. Additional sequences for pancreatic evaluation: CP IRM Coronal radiated T2-weighted thickslice (25 mm) sequences, T2 thin slices MRCP with 3D acquisition | T1-WI, T2-WI, multiphasic dynamic MRI (arterial, portal-venous, and delayed phases) | **-** |  |

| **Somatostatin Receptor Scintigraphy (SRS)** | | | | | | |
| --- | --- | --- | --- | --- | --- | --- |
| Time before acquisition | **-** | Early acquisition at 4h post injection of 111In-pentetreotide and 24h after injection  Or acquisition at 24h and 48h after injection | 24h after injection of 111In-pentetreotide | 111-labeled somatostatin analog performed at 4 to 6 hours and at 24 hours Optional: imaging at 48 hours | Imaging at 4-6 hours and 24-48 hours | **-** |
| Precautions |  | Insulinoma suspicion | Insulinoma suspicion | **-** | **-** |  |
| Preparations |  | Discontinuation of short-acting somatostatin analogs for 24h Long-acting preparations should preferably be stopped 5–6 weeks before Optional: oral laxative | No need for fasting Optional: oral laxative | **-** | **-** |  |
| Dose |  | Dose de 111In-pentetreotide (OctreoScan): 185–222 MBq (5–6 mCi) in adults 5 MBq/kg (0.14 mCi/kg) in children | Dose de 111In-pentetreotide (OctreoScan): 185–222 MBq (5–6 mCi) in adults 5 MBq/kg (0.14 mCi/kg) in children | **-** | **-** |  |
| Image post-processing |  | - | Multiplanar reconstructed images (MPR) 3D maximum intensity projections (MIP) | **-** | **-** |  |

| **Positron Emission Tomography/CT (PET/CT)** | | | | | | |
| --- | --- | --- | --- | --- | --- | --- |
| CT acquisition protocol | **-** | **-** | Low-dose non-enhanced CT for attenuation correction Optional: contrast-enhancement CT | **-** | **-** | **-** |
| Image post-processing |  |  | Multiplanar reconstructed images (MPR) | **-** | **-** | **-** |
| Preparations |  |  | No need for fasting No discontinuation of short-acting somatostatin analogs Examination before long-acting somatostatin analogs injection Optional: oral laxative | **-** | **-** | **-** |
| SSTR PET/CT |  |  | 68Ga-DOTA-TOC/TATE/NOC Dose: 2 MBq/kg BW as a bolus Time before examination: 60 min Examination time: 20 min | **-** | **-** | Dose: 185 MBq (5 mCi) in adults 2 MBq (0.054 mCi)/kg in children |
| 18F-FDG PET/CT |  |  | Dose: 4 MBq/kg BW as a bolus Time before examination: 60 min Examination time: 20 min 4/6-hour fasting period Blood glucose level test | **-** | **-** | **-** |
| **Supplementary Table 1: Extracted relevant data: general**  Abbreviations: ENETS: European Neuroendocrine Tumor Society, WHO: World Health Organization, TNM: Tumor Node Metastasis, UICC: Union for international Cancer Control, CT: computed tomography, MRI: Magnetic resonance imaging, SPECT: single-photon emission computed tomography, PET: positron emission tomography, SSTR: somatostatin receptor, 18F-FDG: Fluorodeoxyglucose, 18F-DOPA: fluorodeoxyphenylalanine, GLP1-R: glucagon like peptide receptor | | | | | | |
