# Supplementary material for: Updated Trends in Imaging Practices for Pancreatic Neuroendocrine Tumors (PNETs): A Systematic Review and Meta-Analysis to Pave the Way for Standardization in the New Era of Big Data and Artificial Intelligence
Source: Front Oncol. 2021 Jul 14;11:628408. doi: 10.3389/fonc.2021.628408 (PMC8316992; doi:10.3389/fonc.2021.628408)
Supplement: Supplementary Table 3 — Search strategies. [file Table_3.docx]

**Supplementary Table 3**

| **Search Strategy for MEDLINE (PubMed)** | |
| --- | --- |
| **Key words** | ((((((("adenoma, islet cell"[MeSH Terms] OR ("adenoma"[All Fields] AND "islet"[All Fields] AND "cell"[All Fields]) OR "islet cell adenoma"[All Fields] OR ("pancreatic"[All Fields] AND "neuroendocrine"[All Fields] AND "tumor"[All Fields]) OR "pancreatic neuroendocrine tumor"[All Fields]) OR (("pancreas"[MeSH Terms] OR "pancreas"[All Fields] OR "pancreatic"[All Fields]) AND ("neuroendocrine tumours"[All Fields] OR "neuroendocrine tumors"[MeSH Terms] OR ("neuroendocrine"[All Fields] AND "tumors"[All Fields]) OR "neuroendocrine tumors"[All Fields]))) OR ("adenoma, islet cell"[MeSH Terms] OR ("adenoma"[All Fields] AND "islet"[All Fields] AND "cell"[All Fields]) OR "islet cell adenoma"[All Fields] OR ("pancreatic"[All Fields] AND "neuroendocrine"[All Fields] AND "tumour"[All Fields]) OR "pancreatic neuroendocrine tumour"[All Fields])) OR (("pancreas"[MeSH Terms] OR "pancreas"[All Fields] OR "pancreatic"[All Fields]) AND ("neuroendocrine tumours"[All Fields] OR "neuroendocrine tumors"[MeSH Terms] OR ("neuroendocrine"[All Fields] AND "tumors"[All Fields]) OR "neuroendocrine tumors"[All Fields]))) OR ("adenoma, islet cell"[MeSH Terms] OR ("adenoma"[All Fields] AND "islet"[All Fields] AND "cell"[All Fields]) OR "islet cell adenoma"[All Fields] OR ("pancreatic"[All Fields] AND "neuroendocrine"[All Fields] AND "neoplasm"[All Fields]) OR "pancreatic neuroendocrine neoplasm"[All Fields])) OR (("pancreas"[MeSH Terms] OR "pancreas"[All Fields] OR "pancreatic"[All Fields]) AND ("neurosecretory systems"[MeSH Terms] OR ("neurosecretory"[All Fields] AND "systems"[All Fields]) OR "neurosecretory systems"[All Fields] OR "neuroendocrine"[All Fields]) AND ("neoplasms"[MeSH Terms] OR "neoplasms"[All Fields]))) OR ("carcinoma, islet cell"[MeSH Terms] OR ("carcinoma"[All Fields] AND "islet"[All Fields] AND "cell"[All Fields]) OR "islet cell carcinoma"[All Fields] OR ("pancreatic"[All Fields] AND "neuroendocrine"[All Fields] AND "carcinoma"[All Fields]) OR "pancreatic neuroendocrine carcinoma"[All Fields])) OR (("pancreas"[MeSH Terms] OR "pancreas"[All Fields] OR "pancreatic"[All Fields]) AND ("carcinoma, neuroendocrine"[MeSH Terms] OR ("carcinoma"[All Fields] AND "neuroendocrine"[All Fields]) OR "neuroendocrine carcinoma"[All Fields] OR ("neuroendocrine"[All Fields] AND "carcinomas"[All Fields]) OR "neuroendocrine carcinomas"[All Fields])) |
| **Publication period** | ("2014/12/01"[PDat] : "2019/12/01"[PDat]) |
| **Search filters** | English[lang]  "humans"[MeSH Terms] NOT "animals"[MeSH Terms:noexp]  NOT Case Reports[ptyp] NOT (Meta-Analysis[ptyp] OR Review[ptyp] OR systematic[sb]) |
|  |  |
| **Search Strategy for Embase** | |
| **Key words** | (pancreatic AND neuroendocrine AND tumor* OR (pancreatic AND neuroendocrine AND tumour*) OR (pancreatic AND neuroendocrine AND neoplasm*) OR (pancreatic AND neuroendocrine AND carcinoma*) OR pancreas) AND islet AND cell AND tumor* |
| **Publication period** | 01-12-2014]/sd NOT [01-12-2019]/sd |
| **Search filters** | [english]/lim NOT ('nonhuman'/de OR 'case report'/de OR 'medical record review'/de OR 'meta analysis'/de OR 'systematic review'/de) |
|  |  |
| **Search Strategy for The Cochrane Library (Cochrane Central Register of Controlled Trials)** | |
| **Key words** | pancreatic neuroendocrine tumor* in Title Abstract Keyword OR pancreatic neuroendocrine tumour* in Title Abstract Keyword OR pancreatic neuroendocrine neoplasm* in Title Abstract Keyword OR pancreatic neuroendocrine carcinoma* in Title Abstract Keyword OR islet cell tumor* in Title Abstract Keyword - (Word variations have been searched) |
| **Publication period** | 01/12/2014 to 01/12/2019 |
| **Search filters** | ∅ |
